# Nummular Eczema as the Initial Extrahepatic Manifestation of Hepatitis C Virus Infection: A Case Report

**DOI:** 10.7759/cureus.89379

**Published:** 2025-08-04

**Authors:** Carlos E Valdez Ramírez, Lizeth Vera de León, Quitzia L Torres Salazar

**Affiliations:** 1 Internal Medicine, Institute for Social Security and Services for State Workers, Mexico City, MEX; 2 Biomedical Sciences, Universidad Juárez del Estado de Durango, Durango, MEX

**Keywords:** extrahepatic manifestations, glecaprevir, hepatitis c, nummular dermatitis, pibrentasvir

## Abstract

Although dermatological manifestations are common in hepatitis C virus (HCV) infection, nummular eczema is rarely reported in this context. We describe the case of a 73-year-old man with a seven-month history of pruritic lesions on the posterior thorax, unresponsive to symptomatic treatment. Physical and dermoscopic examination revealed well-demarcated, coin-shaped plaques with peripheral erythema and fine desquamation. Histopathology confirmed chronic inflammatory dermatitis. Laboratory tests showed elevated transaminases and high HCV viral load. The patient had a history of blood transfusion two decades prior but no prior HCV diagnosis. He was treated with a 12-week course of glecaprevir/pibrentasvir, which resulted in complete resolution of skin lesions and normalization of liver enzymes within one month. No adverse events were recorded. This case highlights the importance of recognizing atypical dermatological signs as possible early indicators of HCV infection and supports the effectiveness of direct-acting antivirals in resolving both hepatic and extrahepatic manifestations.

## Introduction

Nummular eczema, also known as nummular dermatitis or discoid eczema, is a chronic inflammatory dermatosis characterized by pruritic, coin-shaped, exudative plaques, predominantly located on the extremities and trunk [1]. Although it is rarely documented as an extrahepatic manifestation of hepatitis C virus (HCV), its clinical significance lies in its potential association with this chronic infection [2].

The global prevalence of nummular eczema is approximately 0.2% in the general population, with a slightly higher incidence in men and a greater prevalence among individuals over 50 years of age [3]. Its incidence tends to increase during winter months and in colder climates, likely due to environmental factors such as reduced humidity and increased skin dryness.

While HCV is widely recognized for causing progressive hepatic damage, it also induces extrahepatic manifestations in up to 74% of infected patients [4]. The pathophysiology of these manifestations is linked to the ability of the virus to chronically stimulate the immune system, triggering inflammatory and immunologic responses that can affect various tissues, including the skin. Common dermatological manifestations include mixed cryoglobulinaemia, lichen planus, and porphyria cutanea tarda, as well as less frequent conditions such as pruritus, leukocytoclastic vasculitis, and, rarely, nummular eczema [5]. These extrahepatic manifestations may precede the clinical signs of liver disease, underscoring their role in the early diagnosis of HCV.

Despite the availability of direct-acting antiviral (DAA) therapies that have revolutionized HCV treatment, their impact on specific dermatological manifestations, such as nummular eczema, remains underexplored [6]. This article describes a case of nummular eczema as the initial presentation of HCV infection, highlighting the importance of recognizing atypical clinical presentations for timely diagnosis and comprehensive patient management.

## Case presentation

This case involves a 73-year-old male with a history of blood transfusion 20 years prior, although the indication for the transfusion was unknown. The patient presented with persistent generalized pruritus and a localized dermatosis on the posterior thoracic region. The lesions were characterised by well-demarcated, erythematous plaques with regular borders, pruritic in nature, and of chronic evolution over seven months. Despite receiving conventional medical treatment for symptomatic relief, the lesions showed no clinical improvement. After seven months of persistent dermatosis without clinical response, treatment with a 12-week course of glecaprevir/pibrentasvir was initiated. Remarkably, the skin lesions resolved completely within the first month of therapy, accompanied by normalization of liver enzyme levels.

**Figure 1:**
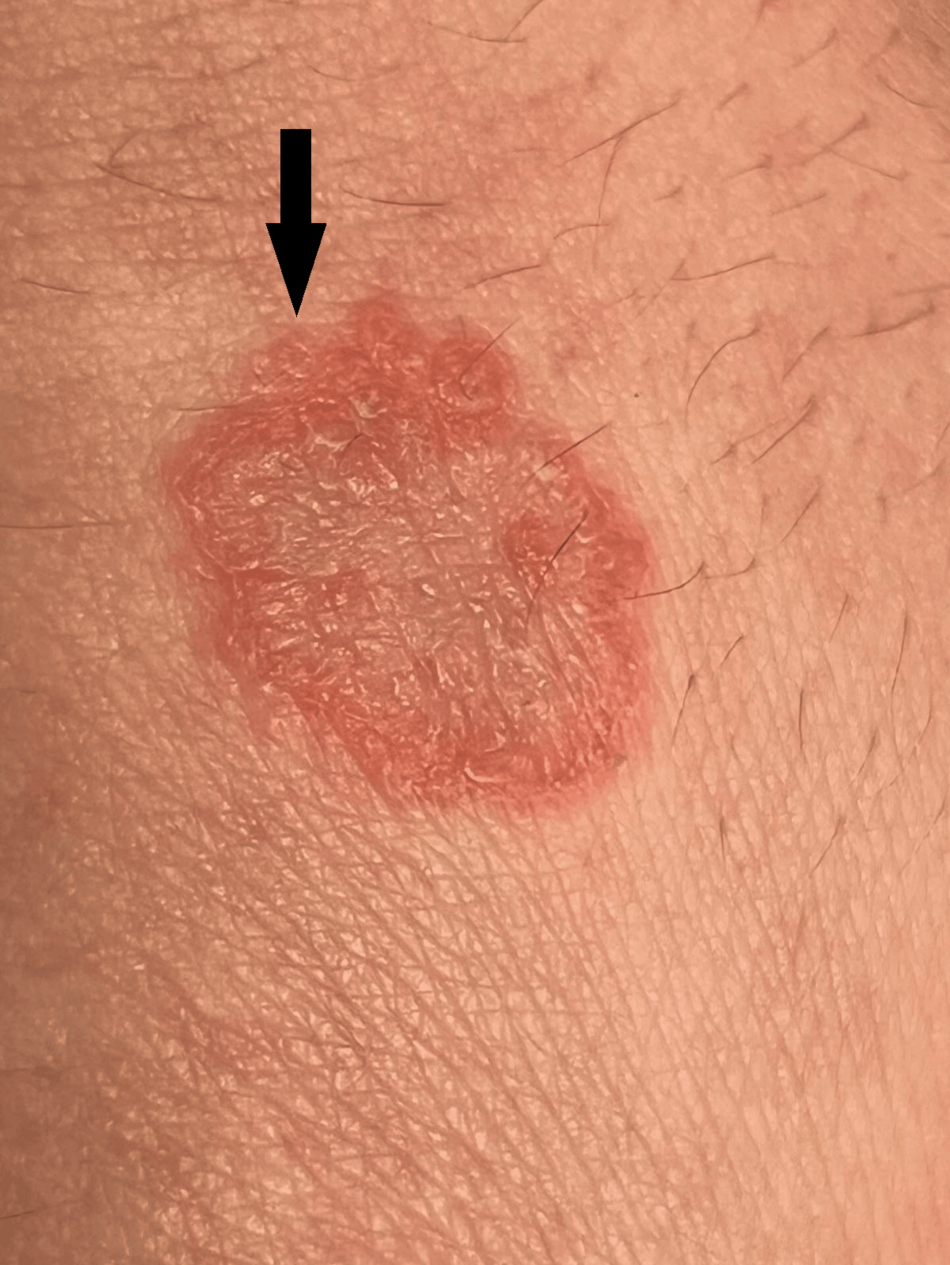
Clinical image of a solitary, well-demarcated, oval lesion (1.5-2 cm) on the posterior thoracic region, showing peripheral erythema, fine scaling, and a central pink, dry area consistent with nummular eczema.

Physical examination revealed a solitary, oval-shaped lesion with well-defined borders and a diameter of approximately 1.5-2 cm. The lesion displayed intense peripheral erythema with mild elevation and fine surface scaling. The central area appeared pinkish, dry, and scaly, with no exudate. No ulcerations, crusts, or pigmentary changes were observed (Figure 1). Initial laboratory tests revealed a fourfold elevation of transaminases above the upper limit of normal, while other parameters were within normal limits (Table 1).

**Table 1:** Laboratory findings at initial presentation.

Parameter	Patient Value	Reference Range
Alanine aminotransferase (ALT)	224 U/L	7–56 U/L
Aspartate aminotransferase (AST)	164 U/L	5–40 U/L
Total bilirubin	0.9 mg/dL	0.1–1.2 mg/dL
Alkaline phosphatase (ALP)	92 U/L	44–147 U/L
White blood cell count (WBC)	7,500 /μL	4,000–10,000 /μL
Platelet count	210,000 /μL	150,000–400,000 /μL
Hemoglobin	14.1 g/dL	13.5–17.5 g/dL (men)
Anti-HCV antibodies	Positive	Negative
HCV RNA viral load	791,000 IU/mL	Undetectable

**Figure 2:**
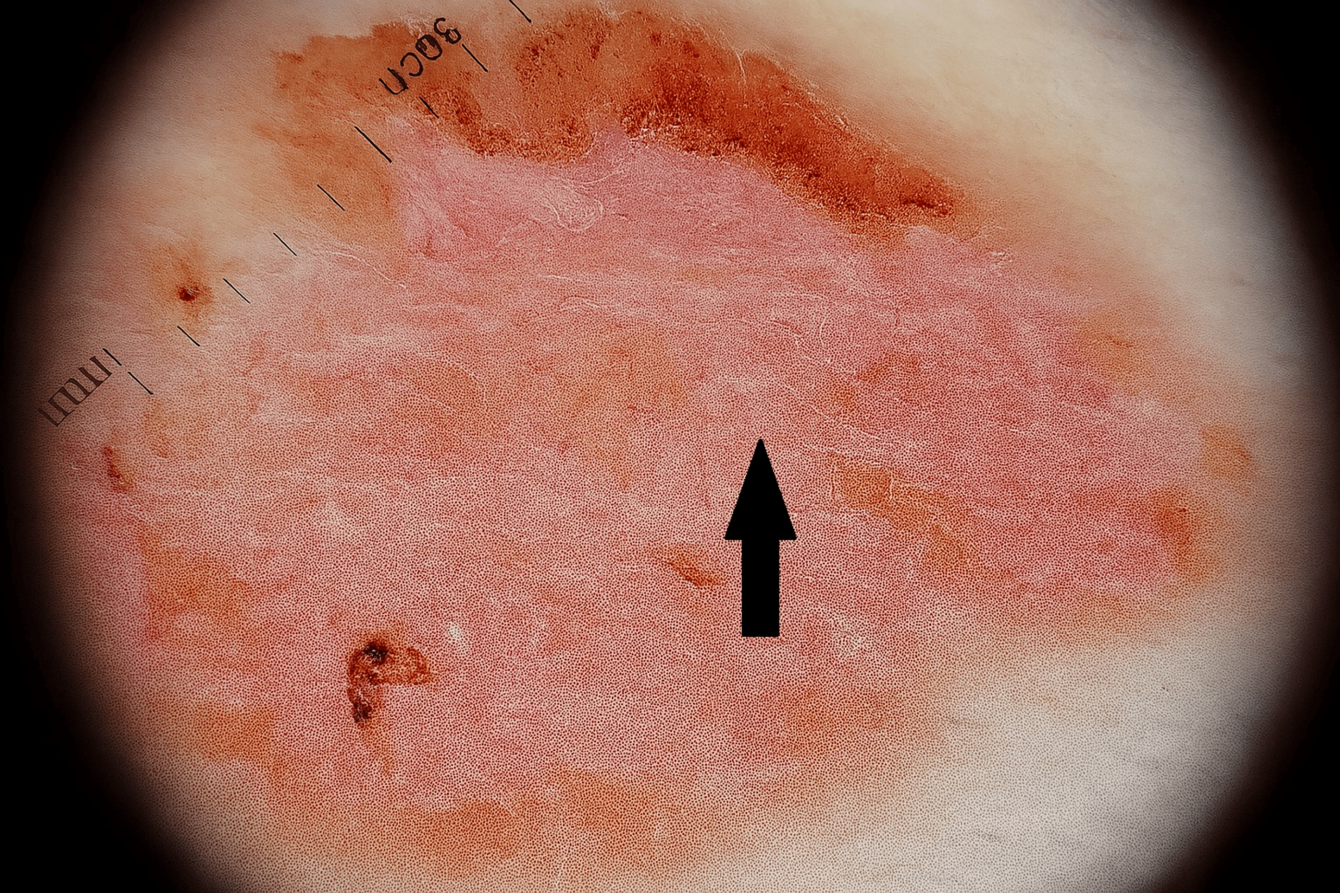
Dermatoscopic image showing a pinkish-erythematous background with irregular superficial desquamation and central dry scaling, compatible with chronic inflammatory dermatosis suggestive of nummular eczema.

Dermatoscopic examination revealed a homogeneous pinkish-erythematous background, with well-defined areas of superficial desquamation with irregular borders. The central region appeared dry and scaly, without significant pigmented structures. This pattern was consistent with chronic inflammatory dermatitis and supported a clinical diagnosis of nummular eczema (Figure 2).

**Figure 3:**
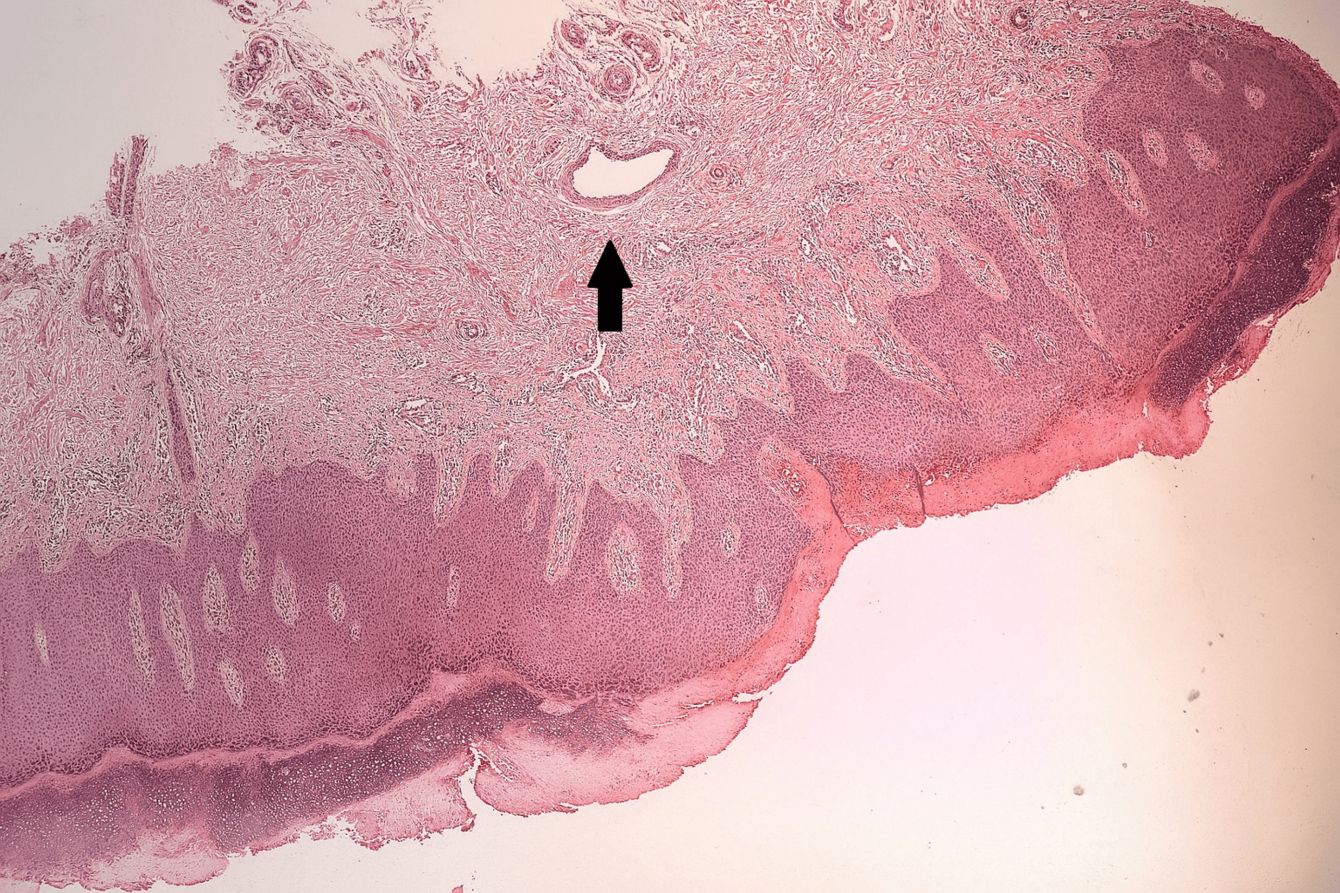
Skin biopsy showing epidermal perinuclear vacuolization, interface dermatitis, and chronic inflammatory infiltrate in perivascular and periappendageal regions.

Histopathological analysis of a skin biopsy revealed epidermal perinuclear vacuolisation, interface dermatitis in the dermis, and a chronic inflammatory infiltrate localized to perivascular and periappendageal regions (Figure 3).

Due to the persistent lesions and elevated transaminases, confirmatory testing for HCV was performed, yielding a viral load of 791,000 IU/mL. A 12-week course of glecaprevir/pibrentasvir (100 mg/40 mg) was initiated. The patient showed significant clinical improvement, with complete resolution of skin lesions within one month and normalisation of transaminase levels. No adverse events were reported during follow-up.

## Discussion

This case describes nummular eczema as an initial extrahepatic manifestation of HCV infection. Although dermatological manifestations in HCV patients are well-documented, reports of nummular eczema in this context remain rare. Previous studies, such as that by Mohammad et al., reported that the most common cutaneous manifestations of HCV infection include pruritus (33.96%), lichen planus (23.5%), and mixed cryoglobulinemic vasculitis (8.49%) [5]. Tawfik et al. [2] documented a 14.3% prevalence of eczema in patients with HCV-related cryoglobulinaemia, suggesting a potential link between HCV-induced immune dysfunction and the development of dermatitis.

The present case is notable in that nummular eczema preceded the HCV diagnosis and was not related to antiviral therapy. This contrasts with reports by Moore et al., where nummular eczema was observed as an adverse reaction to interferon alpha‑2b and ribavirin [6]. In our patient, the most probable source of infection was a blood transfusion received two decades earlier, suggesting a long-standing, undiagnosed HCV infection. Such chronicity may have contributed to sustained immune activation, facilitating the development of extrahepatic cutaneous manifestations. This highlights the importance of considering nummular eczema as an initial extrahepatic manifestation of HCV, particularly in patients with risk factors such as prior blood transfusions.

Additionally, the case differs from those in larger series, such as those by Mohammad et al. and Tawfik et al., which identified cryoglobulinaemia or lichen planus as the predominant cutaneous manifestations [2,5]. Treatment with direct-acting antivirals has been shown to improve dermatological lesions. In this case, therapy with glecaprevir/pibrentasvir resulted in complete lesion resolution, supporting the findings of Tawfik et al. regarding the effectiveness of DAAs in treating skin involvement [2].

The pathogenesis of dermatological extrahepatic manifestations in HCV infection, independent of antiviral therapy, involves chronic immune activation. Persistent infection promotes expansion of autoreactive B cell clones, immune complex formation, and sustained cytokine release (TNF‑α, IL‑6, and IFN‑γ). As highlighted by Del Padre et al., BCR/TLR9 crosstalk in exhausted CD21 low B cells fosters survival and activation even after viral clearance, supporting immune-mediated mechanisms that underlie cutaneous involvement such as nummular eczema. These pathways suggest that prolonged immune dysregulation may sustain extrahepatic dermatologic disease in chronic or longstanding HCV infection [7].

The clinical relevance of this case lies in several aspects. First, it underlines the need for high suspicion of atypical cutaneous signs in patients with HCV risk factors, allowing for early diagnosis and prevention of hepatic complications. Second, it illustrates the effectiveness of antiviral therapy not only in eradicating the virus but also in resolving its extrahepatic sequelae. Finally, it adds to the growing body of evidence suggesting that chronic inflammatory dermatoses may be linked to immune responses triggered by HCV infection.

## Conclusions

Nummular eczema, though rare, may serve as a clinically significant initial extrahepatic manifestation of HCV infection. In this case, the patient’s dermatological presentation preceded the diagnosis of HCV and was unrelated to antiviral therapy, underscoring the importance of recognizing atypical extrahepatic signs in patients with established risk factors such as prior blood transfusion. The resolution of skin lesions following glecaprevir/pibrentasvir not only confirms the effectiveness of DAA in eradicating HCV but also demonstrates their role in reversing associated immune-mediated dermatological manifestations. This highlights the need for a comprehensive diagnostic approach that integrates dermatologic assessment into HCV screening strategies, particularly in patients with chronic, asymptomatic infection. Early identification and treatment may prevent progression to advanced hepatic disease, mitigate extrahepatic complications, and ultimately improve both liver-related and systemic outcomes.
